# The Role of Colonoscopy in the Management of Individuals with Lynch Syndrome: A Narrative Review

**DOI:** 10.3390/cancers15153780

**Published:** 2023-07-26

**Authors:** Valentina D’Angelo, Daniela Rega, Pietro Marone, Elena Di Girolamo, Corrado Civiletti, Fabiana Tatangelo, Francesca Duraturo, Marina De Rosa, Mario de Bellis, Paolo Delrio

**Affiliations:** 1 Division of Gastroenterology and Gastrointestinal Endoscopy, Istituto Nazionale Tumori-IRCCS “Fondazione G. Pascale”, 80131 Naples, Italy; v.dangelo@istitutotumori.na.it (V.D.);; 2 Colorectal Surgical Oncology, Department of Abdominal Oncology, Istituto Nazionale Tumori-IRCCS “Fondazione G. Pascale”, 80131 Naples, Italy; 3 Division of AnatomicPathology and Cytopathology, Istituto Nazionale Tumori-IRCCS “Fondazione G. Pascale”, 80131 Naples, Italy; 4 Department of Molecular Medicine and Biomedical Technology, School of Medicine, University Federico II, 80138 Naples, Italy

**Keywords:** Lynch syndrome, colonoscopy, surveillance, colorectal cancer, incident cancer risk, mismatch-repair deficiency

## Abstract

**Simple Summary:**

The research is being suggested from the observation that the incidence of colorectal cancer in individuals with Lynch Syndrome did not entirely decrease as expected, despite the high quality colonscopies performed in the surveillance programs, according to current Guidelines. The authors would like to highlight that the factors responsible for these results are indipendent of both endoscopy surveillance and adherence to prevention programs of individuals affected by Lynch Syndrome. Probably, the peculiarity of carcinogenesis itself in Lynch Syndrome is responsible for the above mentioned unexpected decrease in incidence of colorectal cancer in individuals with Lynch Syndome. Therefore, rather than continuing to shorten the timing of endoscopic surveillance, other early diagnostic techniques and subsequent prevention strategies should be forecasted in order to allow a more effective and customized endoscopic surveillance of individuals with Lynch Syndome.

**Abstract:**

The history of Lynch syndrome changed definitively in 2000, when a study published in Gastroenterology demonstrated a significant reduction in mortality among individuals with Lynch syndrome who undergo regular endoscopic surveillance. As a consequence of this clinical evidence, all scientific societies developed guidelines, which highlighted the role of colonoscopy in the management of Lynch syndrome, especially for individuals at high risk of colorectal cancer. Over the years, these guidelines were modified and updated. Specialized networks were developed in order to standardize endoscopic surveillance programs and evaluate all the clinical data retrieved by the results of colonoscopies performed for both the screening and the surveillance of individuals with Lynch syndrome. Recent data show that the impact of colonoscopy (with polypectomy) on the prevention of colorectal cancer in individuals with Lynch syndrome is less significant than previously thought. This narrative review summarizes the current discussion, the hypotheses elaborated and the algorithms depicted for the management of individuals with Lynch Syndrome on the basis of the recent data published in the literature.

## 1. Introduction

Lynch syndrome (LS) is an autosomal dominant disorder caused by heterozygous germline mutations in DNA-mismatch repair (MMR) genes, including the MLH1, MSH2, MSH6, PMS2, and EPCAM genes. The latter cause the epigenetic silencing of MSH2. Lynch syndrome presents high penetrance and variable expressivity. Patients with LS have an increased risk of developing colorectal cancer (CRC), with an estimated lifetime risk of 35–80% [1].

In LS, both accelerated progression through the adenoma–carcinoma sequence, and carcinoma arising from non-neoplastic crypt foci are due to MMR deficiencies, which compromise the ability to repair base-pair mismatches in DNA and induce the onset of colorectal cancer (CRC) [2]. Thus, cancers arising in LS exhibit the molecular phenotype of microsatellite instability (MSI) [1,2]. When indel mutations hit coding microsatellites (cMSs), two possible biologically relevant consequences follow: first, mutations in cMSs can lead to the inactivation of tumor-suppressor genes, contributing to carcinogenesis; and second, such mutations shift the reading frame and lead to the generation of frameshift peptides (FSP), rendering MSI tumors highly immunogenic [3].

The LS phenotype is characterized by a predominance of colonic lesions with non-polypoid morphology, proximal colonic location, a higher proportion of high-grade dysplasia (HGD), which is often detected in small lesions, and a predisposition for both synchronous and metachronous CRCs [1]. Therefore, it is necessary to remove all visible lesions that can potentially be “aggressive” adenomas at the time of colonoscopy. This is the rationale for the endoscopic surveillance used in this population.

The history of LS changed definitively in 2000, when the results of a Finnish study were published in Gastroenterology [4]. For the first time, a prospective study was able to prove a significant reduction in mortality among members of 22 Finnish families with LS undergoing regular endoscopic surveillance. The occurrence of incident colorectal cancer between the study group and the control group was statistically significant and none of the cancers diagnosed in the study group were at an advanced stage. None of the 133 subjects in the study group died of CRC, while 9 out of 119 (8%) of the control subjects perished from the disease (*p* < 0.001) [4].

As a consequence of these important results, all scientific societies developed guidelines for the management of individuals with LS, which have been modified and updated over the years [5].

This narrative review summarizes the ongoing discussion, the hypotheses elaborated and the algorithms depicted for the management of individuals with LS on the basis of the recent data published in the literature.

## 2. Four Crucial Points 

Gastroenterology and oncology societies have published guidelines (GLs) for screening and follow-up of subjects with LS who are at high risk for CRC. The suggested endoscopic follow-up times were different in the GLs of the various scientific societies, and these continued to be changed over time in an effort to improve the effectiveness of endoscopic surveillance [6,7,8,9]. Because of the multiplicity of these GLs, some controversial points began to emerge [10]_._

In more recent years, specialized networks were developed to standardize endoscopic surveillance programs. For the first time, it was possible to evaluate a large number of clinical data resulting from surveillance colonoscopies of subjects with LS. These recent data show that the efficacy of colonoscopy with polypectomy for the prevention of CRC in subjects with LS is probably less significant than was previously thought [11]. Based on the results of numerous prospective and retrospective studies, it is now clear that regular colonoscopy screening does not entirely prevent CRC. 

Some crucial points were assessed.

First, the quality of colonoscopy (defined as cecal intubation rate, adenoma/polyp detection rate (ADR > 30% for expert endoscopists, according to ESGE), complete polyp resection, and bowel cleanliness) has been evaluated in various trials and meta-analyses [12]_._ Recent data show that the quality of colonoscopy alone does not explain the occurrence of incident CRC [13]. Quality scores for colonoscopy and polypectomy are important, but they are not capable of counteracting the celerity of non-adenoma pathways in subjects with LS. Chromoendoscopy probably does not improve the efficacy of colonoscopy in subjects with LS; on the other hand, this technique overdetects hyperplastic polyps [13]. To date, Artificial Intelligence (AI) has not improved the results of high-quality colonoscopy [14]_._ Recently, the detection of a diminutive adenoma by AI in a subject with LS was reported [15]_._ Current AI systems have not been validated in individuals with LS, but the technology certainly holds promise for this high-risk population [16]_._ Until we are able to fully understand the time frame of the non-adenoma sequence, we must improve our ability to identify the lesions characteristic of LS, which are often already invasive, despite their small size. In fact, the general recommendation for LS surveillance is to remove all detected lesions that can potentially be aggressive adenomas: this is the rationale for the current annual/biennial-surveillance strategy in MMR-mutation carriers. The small size, the non-polypoid morphology, and the proximal colon location of the lesions are all possible causes of misdiagnosis by endoscopists. The current ESGE guidelines recommend high-definition white-light colonoscopy for the surveillance of LS individuals [7]. Nonetheless, there is a need to develop better image-enhanced endoscopy to increase the effectiveness of colonoscopy surveillance in LS individuals who remain at high risk. To further explore these aspects, several studies have investigated the application of a new technique for the enhancement of endoscopic images, the usefulness of AI for the surveillance of LS individuals, and the efficacy of the “diagnose and leave in” strategy in LS.

A recent prospective, randomized trial analyzed the efficacy of (LCI) equipment-based image-enhanced endoscopy in 700 patients of the general population, excluding those with hereditary syndromes [17]. Linked color imaging equipment-based image-enhanced endoscopy was compared with white-light imaging (WLI) by assessing their respective detection (ADR) and the miss (AMR) rates for adenomas. The LCI method improved the average ADR of only low-detector endoscopists compared with high detectors (*p* < 0.001). On the other hand, LCI significantly lowered the AMR, especially in the case of diminutive adenomas and non-polypoid lesions with a diagnostic improvement, even for expert endoscopists with high ADRs. The results of this study imply that LCI could be used for the detection of lesions that are characteristic of LS, which are often misdiagnosed.

In 2022, a randomized controlled pilot trial evaluated the efficacy of artificial-intelligence-assisted colonoscopy in the surveillance of carriers of pathogenic germline variants (MLH1, MHS2, MSH6) [18]. This study explored AI-assisted colonoscopy vs. HD white-light colonoscopy. There was a higher detection rate for flat and completely flat adenomas (II-a and II-b sec. Paris classification) using AI, and similar durations of colonoscopy. The risk of the increased detection of clinically irrelevant lesions was excluded by the reported absence of significant differences in the number of hyperplastic lesions diagnosed in both groups. These results did not reach statistical significance due to the small sample size, but they certainly reflected the real-world surveillance scenario and suggested the need for further studies. 

A recent randomized controlled trial resumed the discussion about the unnecessary overtreatment of irrelevant lesions detected during the surveillance colonoscopies of individuals with LS [19]. According to the ASGE-PIVI’s first statement regarding the “diagnose and leave in” strategy for diminutive rectosigmoid polyps, the negative predictive value (NPV) for adenomatous histology must not be inferior to 90% when used with a high level of confidence to introduce new strategies into current clinical practice [20].

Out of one hundred and twenty-eight diminutive (≤5 mm) rectosigmoid lesions diagnosed in vivo by real-time optical diagnosis, with virtual chromoendoscopy alone or with dye-based chromo-endoscopy, only three neoplastic lesions were wrongly classified as hyperplastic polyps and eight non-adenomatous lesions were misdiagnosed as neoplastic. Thus, the use of NPV for neoplastic histology among diminutive rectosigmoid lesions exceeded the benchmark of 90%, indicating both the feasibility and the safety of the “diagnose-and-leave-in” strategy for the surveillance of individuals with LS [19]. The results of this study suggest the possibility that enhanced endoscopy may improve the characterization of colorectal lesions in the LS population. However, these findings need to be verified in further studies, which should also clarify whether it is acceptable to leave in place even one diminutive neoplastic polyp in the rectosigmoid colon. Finally, several detrimental aspects of colonoscopy were addressed in another study [21]. These included spasms of the sigmoid colon, severe diverticulosis with a fixed and angulated sigmoid colon, painful colonoscopy limiting repeated inspection of some areas of the colon, and unstable patients with severe bradycardia, hypotension, or decreased oxygen saturation [21]. All these crucial factors are not taken into account in the definition of the recommended intervals between two subsequent surveillance colonoscopies. A proposed new subjective quality score, referred to as the ‘Leiden Quality Score’, is based on the endoscopist’s overall impression of the quality of the colonoscopy and his/her subjective polyp-miss rate [21]. This score could be especially important with LS because the surveillance interval may be shortened, according to the ‘Leiden Quality Score’.

The second point is that cancer-risk education is necessary, but it is challenging. Subjects with LS need clear and repeated explanations about the value of endoscopic surveillance. Often, they also require psychosocial support. The usefulness of specialized programs aiming to remind patients of the dates of both exams and clinical follow-ups has been demonstrated [22]_._ Recent studies argued that prevention can still fail: a significant number of subjects with LS develop CRC [11,23,24,25]. This means that non-compliance may not explain the majority of CRC cases diagnosed in subjects with LS [14]. However, patient compliance is still considered crucial for successful cancer-screening programs. Several studies focused on breast, cervical, and colorectal cancer screening, evaluating social determinants, such as economic disadvantage, lower education, psychological difficulties (both personal and familial), and ethnic-minority status, in order to plan support strategies to improve the success of cancer-screening programs. These strategies are mainly composed of patient-navigation programs [26]. 

The results of a recent randomized trial suggest that a patient-navigation program could be effective for improving colorectal cancer screening among first-degree relatives of subjects affected by LS who are at high and immediate risk of colorectal cancer [27]. 

In this study, two different ways to recommend colorectal cancer screening were compared. Members of families at risk of colorectal cancer were randomized to either receive website intervention alone or website-plus-patient-navigation intervention. Of 513 families, 259 were randomized to the website-intervention arm, while 254 families were randomized to the website-plus-patient-navigation intervention arm. A document containing personal colorectal-cancer screening recommendations and the date of the colonoscopy was delivered online and/or mailed/emailed. This document also included suggestions about healthy lifestyles: correct diet, good quality of sleep, adequate physical activity, and smoking cessation. These recommendations were linked to the information regarding colorectal cancer from institutional websites: NCI, the American Cancer Society, and the American Gastroenterological Association. In addition to the personal document with access to the website, the participants assigned to the arm of combined intervention received telephone calls from patient navigators. Through multiple calls, the navigators assessed barriers to screening, provided counseling to remove these barriers, and assisted participants with the scheduling of their colonoscopy. Nearly 80% of all the participants in both arms were adherent to the intervention without any differences based on the type of strategy, with clear success demonstrated by both. However, the addition of the patient navigator to a website intervention was crucial to obtain the best adherence to colorectal screening among the first-degree relatives of individuals with LS [27].

Similarly, an ongoing trial, whose protocol was recently published, has the goal of finding the best way to ameliorate the communication of genetic-test results to members of families at high risk of colorectal cancer and to improve the adherence of family members to screening and surveillance programs [28].

The assumption at the basis of this study is that genetic testing itself does not improve the outcomes of cancer screening and surveillance in hereditary cancer, which can be implemented only by the strict adherence of subjects with LS to both screening and follow-up. In this setting, communication plays a very important role. The goal of this study is to evaluate the effectiveness of real-life approaches that can optimize both family communication and guideline-based cancer-risk management. 

The third point is that specialized networks associated with endoscopic surveillance programs have been developed. They aim to standardize surveillance programs and evaluate their effectiveness [29]. It seems unlikely that inter-observer differences or technical limitations are the only explanations for the high proportion of incident cancer detected in subjects undergoing regular colonoscopic surveillance. Different timings of surveillance colonoscopies did not affect the incidence, stage, and prognosis of CRC (Table 1) [11]. The increased frequency of colonoscopy surveillance was a “choosing wisely” strategy, but this has not been supported by scientific evidence [13].

The fourth point is that recent data suggest that when using the GLs for LS, the specific cancer risk on the basis of the different genes involved in LS should be considered (Table 2) [31,34]. The correct definition of MMR genetic variants is crucial to establish the proper interval of surveillance colonoscopy, since the pathogenicity of each MMR genetic variant is significantly different. Accordingly, clinicians can choose not only the most appropriate endoscopic surveillance program but also the most precise medicine [35]. Recently, a decision analytic model established the application of an algorithm for each of the four pathogenetic variants (PV) of LS [36]. The goal was to determine whether the optimal CRC surveillance strategies had to be different for different carriers of different PVs. At the same time, a cost-effectiveness analysis was carried out to evaluate the costs of surveillance, cancer care, and colonoscopy complications. Lifetime cancer incidence and cancer mortality rates are significantly divergent in different PVs, and this can lead to different endoscopic surveillance programs. A less aggressive form of surveillance is recommended for MSH6 and PMS2 carriers, with the first colonoscopy executed at 35 and 40 years of age, respectively, unless there is a family history of earlier cancers. Subsequently, both groups of subjects should undergo endoscopic surveillance every 3 years. Carriers of MLH1 and MSH2 should be monitored according to the current recommendation, with a colonoscopy every 1–2 years, starting at 25–30 years of age [26,36]. A recent population-based study confirmed these recommendations, showing that MSH6 and PMS2 carriers have a significantly lower risk of metachronous colorectal cancers than MLH1 and MSH2 carriers [37]. The aggressive surveillance recommended by the widely applied current guidelines is associated with lower quality-adjusted life-years (QALYs) for subjects with LS [38]. This highlights the importance of a gene-specific approach to CRC surveillance in subjects with LS, with less aggressive strategies, considering that these patients may undergo more than 50 colonoscopies in their entire life [38]. Future studies are needed to understand both the acceptability and the feasibility of new and diversified approaches.

## 3. Some Points of Clarification

The higher incidence of CRC among LS carriers, despite proper endoscopic surveillance and high-quality colonoscopy, raised the question of whether cancers arise rapidly through a non-adenoma pathway. Some hypotheses have been developed for the diagnosis of incident CRC in LS despite regular colonoscopy [39]. A recent study evaluated the hypothesis that incident CRC (found during follow-up colonoscopies) may be phenotypically different from prevalent CRC (found on the first colonoscopy) in subjects with LS [32]. The clinical, histological, and molecular characteristics of incident CRCs were compared with those of prevalent CRCs. The T stage of incident cancers was significantly lower than that of prevalent CRCs, and no T4 lesions were identified among incident cancers. The majority of incident CRCs developed after a colonoscopy in which no lesions were detected [32]. The results of this trial confirm the findings reported in previous studies: incident CRCs often have a mucinous component, a low T stage, with no lymph-node involvement, and a favorable clinical course in subjects with LS [25,30,32,40,41,42,43]. This is not attributable to a potential low-quality colonoscopy. 

At the molecular level, a higher proportion of APC mutations was found in incident CRCs compared to prevalent CRCs in subjects with LS. These mutations were significantly associated with signatures of MMR deficiency compared with prevalent CRCs [29]. On the other hand, KRAS mutations were less frequent than expected [30]. Two hypotheses have been proposed to explain the observed differences. Incident cancer may represent a distinct early event, in which MMR-D commonly precedes APC mutations and the endoscopic removal of precursor lesions during surveillance may be more effective in preventing KRAS-mutated lesions [44], since KRAS mutations are associated with conventional adenomas [45]. This implies that incident cancers may develop from other lesions that are more difficult to detect. Alternatively, incident and prevalent cancers could be two entities representing manifestations of the same pathway, detected at an early or late stage [46]_._

Other authors retrospectively evaluated the clinicopathological characteristics of both adenomas and early cancers that were endoscopically resected in subjects with LS and individuals without LS, respectively [33]. The malignant potential of these neoplastic lesions was analyzed as a function of their location, size, and morphology. High-grade dysplasia (HGD) adenomas smaller than 5 mm were analyzed for MMR protein expression by immunohistochemistry (IHC) to confirm whether small HGD adenomas had deficient MMR protein levels. The malignant transformation intervals from low-grade dysplasia (LGD) adenomas to HGD/early cancerous lesions were homogenous throughout the colon in subjects with LS, in contrast with unaffected individuals [46]. It is known that in the general population, the speed of the growth of colonic lesions has different genetic and/or microbiota-related backgrounds in the proximal and in the distal colon, respectively [33]. This study highlighted the possibility that small HGD adenomas have deficient MMR protein levels, and this was supported by the finding that only 15% of early colonic cancers < 10 mm diagnosed in the study population had focal adenomatous tissue [46].

## 4. Open Questions 

These hypotheses raise several questions: assuming that there is more than one pathway to CRC, what is the relative contribution of each? Are there different genetic backgrounds? Which are they?

A higher incidence of CRC is observed at shorter intervals than expected in subjects with LS [47]. This is surprising; could it be explained by the spontaneous disappearance of colonic lesions? The low probability of lymph-node and distant metastases and the good prognosis of LS-related CRCs may also reflect the immune system’s ability to restrain CRC [47]. The intriguing possibility that some CRCs may spontaneously disappear raises the following question: could it be possible that the host immune response not only removes CRC precursors but also eliminates some infiltrating cancers? Several years ago, MSI-induced frameshift peptides (FSPs) were identified as targets of cellular immune responses in MSI-H tumor patients and in healthy LS germline mutation carriers [48]. Subsequently, the frameshift neoantigen paradigm was defined, according to which dMMR CRCs are characterized by high levels of tumor mutation; this DNA instability leads to an abundant load of mutation-derived neoantigens that trigger a robust immune response in the tumor microenvironment with tumor-infiltrating lymphocytes. It was immediately clear that d-MMR cancers are an ideal tumor model to study the onset, development, and evolution of cancers, along with the immune response occurring during the entire process of carcinogenesis. Currently, immunotherapy is emerging as the most significant revolution in cancer treatment since the development of chemotherapy in the 1940s. The clinical impact of immuno-prevention is potentially even more significant than that of immunotherapy, since early cancers induce less immunosuppression than advanced tumors. In the last 15 years, published studies about immune system’s ability have extended the knowledge about tumor microenvironment, with different options emerging as a result. These include cancer vaccines, cell-based therapy, immune check- point blockade, and oncolytic-virus-based therapy. Thus, the necessity of coordinated and integrated multidisciplinary approaches arose, which led to the “Immuno-Oncology Translational Network” (IOTN) [49]. The IOTN is a task force of 32 academic institutions in the USA, which aims to develop translational research in cancer immunotherapy, personalized immuno-oncology approaches for patients with cancer, and strategies of cancer prevention. 

Recently, some models of carcinogenesis particular to LS have been proposed [44,50]. An experimental model regarding the MSH2 and PMS2 pathogenetic-variant carriers speculates that APC and KRAS mutations may represent the starting events, and that the adenoma is the precursor of the cancer. The MMR deficiency (MMR-D) occurs before or during LS carcinogenesis, accelerating the carcinomatous transformation of the adenoma [44,50]. This model may explain up to 25% of the CRCs in LS.

Another model instead hypothesizes that the lesions originate directly against a background of MMR-D. The MMR-DCF (Deficient Crypt Foci) is a colon crypt that is undetectable by endoscopy and with normal histology, but already lacks MMR protein expression. The gut surface appears normal before the formation of the lesion, but this is already an adenocarcinoma, although it is small in size when it originates [44,50]. This model could explain up to 75% of the CRCs in MLH1 and MSH2-pathogenetic-variant carriers. 

Approximately one MMR-DCF per cm^2^ of mucosa has been observed in phenotypically normal intestinal mucosa. Most of these lesions do not seem to progress to malignancy, as suggested by the discrepancy between the large number of MMR-DCFs and the relatively small number of adenomas or carcinomas observed in LS carriers. The lesions originating from MMR-DCF can grow under intact mucosal surfaces and directly evolve into manifest cancer, without macroscopically visible non-invasive precursors [44,50]. This model may explain up to 10% of the CRCs in MLH1-pathogenetic-variant carriers.

Unlike the well-established germline pathogenic variants in MMR, which help to identify LS, much less is known about the acquired molecular alterations arising in the precursors of CRC, which may serve as early detection markers for both screening and surveillance. While colonoscopy is the best available screening test for CRC in the general population, individuals with LS need accurate and less invasive surveillance tests. Indeed, there are two specific problems: the peculiar pathways of LS carcinogenesis and the elevated lifetime risk of CRC, respectively. A possible solution may be the identification of blood and/or fecal biomarkers, which could be used to establish the proper timing of operative colonoscopy. The ideal characteristics of a biomarker involve a compromise between high sensitivity and accurate specificity, good patient acceptability, a simple sampling technique, rapid data analysis, and cost-effectiveness [51]. Individuals with LS need new diagnostic tests to reduce the number of colonoscopies they undergo and to customize the timing of their endoscopic surveillance. To identify effective fecal markers, cancer DNA, and RNA, metabolomics and gut-microbiome composition should be investigated [51,52]. The aberrant biology of Lynch-associated colorectal neoplasia represents a perfect framework for designing and performing such research. 

A retrospective study was published that identified methylated DNA markers (MDMs) for the detection of LS colorectal cancer by means of next-generation methylation sequencing [53]. The MDMs achieved high efficacy for advanced adenomas and CRCs in both LS and sporadic colorectal tissues. The authors believe that these represent possible tools for the early detection of CRC in LS individuals, complementing current screening strategy [53]. These MDMs are biomarkers that could be part of the surveillance armamentarium and they may be used for testing the stool and/or blood of this high-risk group to prevent interval cancer (Table 3). Future studies may develop a panel of specific markers for LS, which could potentially be used to complement colonoscopy.

## 5. Conclusions 

Colonoscopy has failed to prevent CRC in individuals with LS. Is this in conflict with the results of the previous Finnish follow-up study? The answer is no.

**Figure 1:**
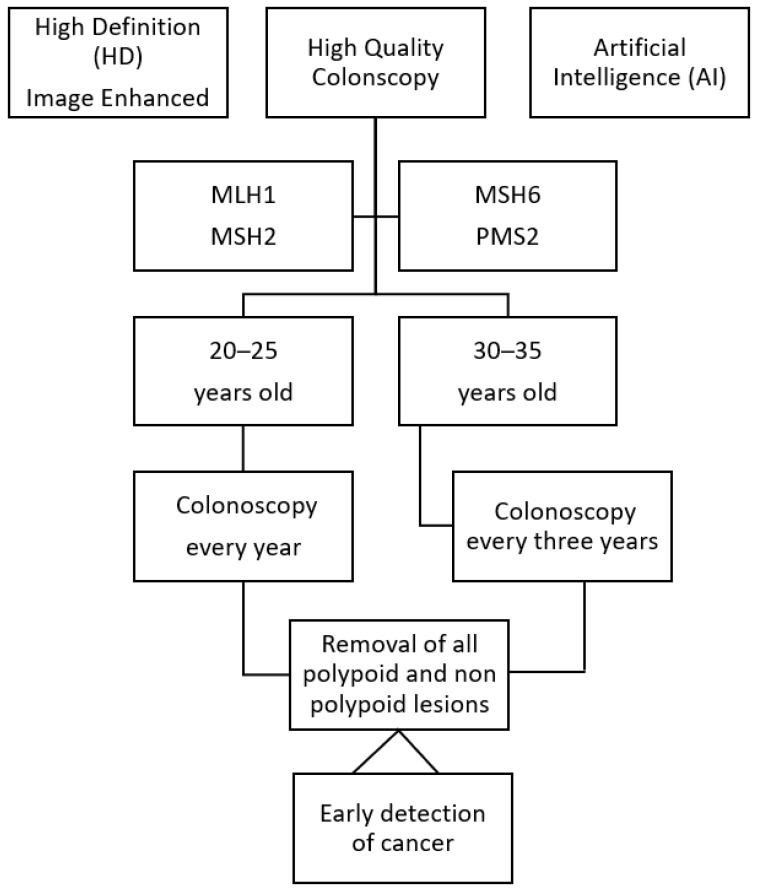
Current surveillance protocol for individuals with Lynch syndrome.

**Figure 2:**
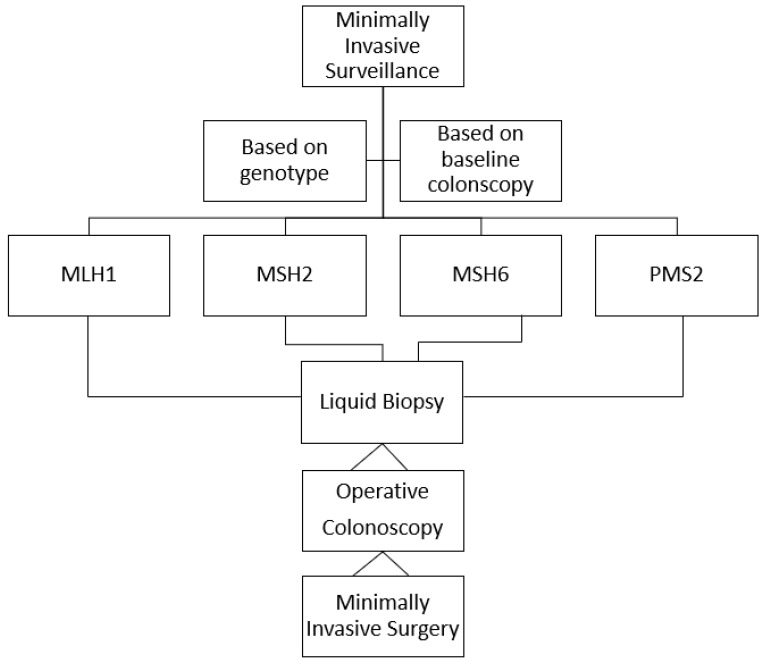
Proposed future surveillance protocol for individuals with Lynch syndrome.

The survival pattern of individuals with LS has undoubtedly changed over generations. The survival rate is not correlated with the interval between colonoscopies and the 10-year survival after CRC is greater than 90% [11]. Colonoscopy remains the central pillar of cancer prevention in LS, with a reported risk of severe complications of 0.3%, and it is administered according a current protocol that is based on the genotypes of individuals with LS [39] (Figure 1). Currently, MMR-pathogenetic variant carriers rarely succumb to CRC or endometrial cancer, while there is an increased incidence of other LS cancers, which occur later in life. These are often the causes of the deaths of individuals with LS, since they are difficult to prevent and cure [11]. Therefore, there is a need to move towards personalized, minimally invasive surveillance, which should feature liquid biopsies of peripheral blood. The latter will establish the need and the timing of operative endoscopy followed by minimally invasive surgery when needed, while allowing the early diagnosis of extraintestinal cancers (Figure 2). 

## 6. Future Directions

To continuously ameliorate the prevention strategy for individuals with LS, we need to critically review the current guidelines and set up a panel of specific markers for the surveillance of individuals with LS.

## Figures and Tables

**Table 1:** Cancer risk and timing of surveillance colonoscopy.

Authors	Study Type	Numbers	Results
Dove-Edwin I et al. [23]	Prospective observational 2005	1678 patiens	Interval colonoscopy on the basis of family history
Engel C et al. [30]	Prospective coohrt 2010	1126 patients	Annual colonoscopy is recommended
Lindberg LJ et al. [31]	Prospective observational 2017	13,444 surveillance sessions	Elevated cancer risk despite biannual surveillance
Engel C et al. [25]	Population 3 countries 2018	2747 patients	Similar cancer risk at different intervals of surveillance colonscopy
Ahadova A et al. [32]	Prospective 2021	28 incident cancer 7 prevalent cancer	Timing of colonoscopy does not modify cancer risk
Bultman SJ [33]	Retrospective 2021	325 removed lesions	Malignant-transformation interval is similar in all segments of the colon

**Table 2:** Cancer risk on the basis of specific gene mutation.

Authors	Study Type	Patients Enrolled	Results
Møller P et al. [11]	Prospective observational 2017	6350	Cancer risk related to specific gene mutation and gender of individuals with LS
Dominguez-Valentin M, et al. [24]	Prospective observational 2020	6350	Cancer risk related to specific gene mutation, gender, and age of individuals with LS
Kastrinos F et al. [36]	Simulation model 2021	6350	CRC surveillance intervals on the basis of the type of specific gene mutation

**Table 3:** Fast progression and early detection of colorectal cancer.

Authors	Study Type	Patients Enrolled	Results
Argillander TE et al. [42]	Retrospective 2018	905	Fast progression of adenoma ≥ carcinoma sequence
Ballester V et al. [53]	Sequential case–control 2020	53	Potential use of MDMs (methylated DNA markers in screening and surveillance)

## Abbreviations

CRC (colorectal cancer), LS (Lynch syndrome), MMR-D (mismatch-repair deficiency), ADR (adenoma-detection rate), GLs (guidelines), PLSD (prospective Lynch syndrome database), ESGE (European Society for Gastrointestinal Endoscopy).
